# Targeting SOX4/PCK2 signaling suppresses neuroendocrine trans-differentiation of castration-resistant prostate cancer

**DOI:** 10.1186/s13062-024-00500-2

**Published:** 2024-07-16

**Authors:** Nan Jing, Zhenkeke Tao, Xinxing Du, Zhenzhen Wen, Wei-Qiang Gao, Baijun Dong, Yu-Xiang Fang

**Affiliations:** 1 State Key Laboratory of Systems Medicine for Cancer, Renji-Med-X Stem Cell Research Center, Ren Ji Hospital, School of Medicine, Shanghai Jiao Tong University, Shanghai, 200127 China; 2 School of Biomedical Engineering and Med-X Research Institute, Shanghai Jiao Tong University, Shanghai, 200030 China; 3 Department of Urology, Ren Ji Hospital, School of Medicine, Shanghai Jiao Tong University, Shanghai, 200127 China

**Keywords:** SOX4, PCK2, Neuroendocrine prostate cancer, Carbohydrate metabolism reprogramming

## Abstract

**Background:**

Neuroendocrine prostate cancer (NEPC), a lethal subset of prostate cancer (PCa), is characterized by loss of AR signaling and resistance to AR-targeted therapy. While it is well reported that second-generation AR blockers induce neuroendocrine (NE) trans-differentiation of castration-resistant prostate cancer (CRPC) to promote the occurrence of NEPC, and pluripotent transcription factors might be potential regulators, the underlying molecular mechanisms remain unclear.

**Methods:**

We analyzed the data from public databsets to screen candidate genes and then focused on SOX4, a regulator of NE trans-differentiation. The expression changes of SOX4 and its relationship with tumor progression were validated in clinical tumor tissues. We evaluated malignant characteristics related to NEPC in prostate cancer cell lines with stable overexpression or knockdown of SOX4 in vitro. Tumor xenografts were analyzed after inoculating the relevant cell lines into nude mice. RNA-seq, ATAC-seq, non-targeted metabolomics analysis, as well as molecular and biochemical assays were carried out to determine the mechanism.

**Results:**

We screened public datasets and identified that expression of SOX4 was significantly elevated in NEPC. Overexpressing SOX4 in C4-2B cells increased cell proliferation and migration, upregulated the expression of NE marker genes, and inhibited AR expression. Consistently, inhibition of SOX4 expression in DU-145 and PC-3 cells reduced the above malignant phenotypes and repressed the expression of NE marker genes. For the in vivo assay, we found that knockdown of SOX4 inhibited tumor growth of subcutaneous xenografts in castrated nude mice which were concomitantly treated with enzalutamide (ENZ). Mechanically, we identified that one of the key enzymes in gluconeogenesis, PCK2, was a novel target of SOX4. The activation of carbohydrate metabolism reprogramming by SOX4 could promote NE trans-differentiation via the SOX4/PCK2 pathway.

**Conclusions:**

Our findings reveal that SOX4 promotes NE trans-differentiation both in vitro and in vivo via directly enhancing PCK2 activity to activate carbohydrate metabolism reprogramming. The SOX4/PCK2 pathway and its downstream changes might be novel targets for blocking NE trans-differentiation.

**Supplementary Information:**

The online version contains supplementary material available at 10.1186/s13062-024-00500-2.

## Background

To date, second-generation androgen receptor pathway inhibitors (ARPIs) such as abiraterone or enzalutamide (ENZ) have been widely used in the treatment of patients with castration-resistant prostate cancer (CRPC) [1, 2]. However, long-period androgen deprivation including the use of ARPIs will usually lead to the emergence of a highly aggressive CRPC subtype, known as therapy-induced neuroendocrine prostate cancer (NEPC), which is characterized by the loss of androgen receptor (AR) expression, the emergence of neuroendocrine features and increased invasion and metastasis [3–5]. Notably, the high expression of neuroendocrine-related proteins like synaptophysin (SYP), neuron-specific enolase (NSE) and chromogranin A (CHGA) can be observed during the process of NE trans-differentiation in multiple cancers [6, 7]. In this context, the identification of key upstream regulators responsible for NE trans-differentiation will be important for future therapeutic optimization.

Besides the dysregulation of several well-known NE-related pathways like the Aurora kinase pathways [8] and the PI3K/Akt/mTOR pathway [9, 10], recent studies also showed that some pluripotent transcription factors such as OCT4 [11] and NANOG [12], can work as upstream regulators of the NE trans-differentiation. On the other hand, more attention has been paid to characterizing the metabolic dysregulation profile during the process of NE trans-differentiation. For example, it has been reported that neuroendocrine tumors contain a higher abundance of oxidized lipids [13]. In addition, activation of proline synthesis also leads to the emergence of NE-like profiling [14].

In the case of prostate cancer, a shift in energy metabolism from oxidative phosphorylation to aerobic glycolysis as the primary energy source usually occurs during the process of NE trans-differentiation [15]. Our previous work also revealed that the PRKAR2B-HIF-1alpha loop promotes aerobic glycolysis for tumor growth in CRPC [16]. However, whether and how pluripotent transcription factors have effects on metabolism reprogramming, particularly through enhancing carbohydrate metabolism reprogramming, to promote NE trans-differentiation, remains unclear.

SOX4, a member of the SRY-related high mobility group (HMG) box transcription factor (TF) family, has been well-reported to function as a pluripotent and stemness-related TF, regulating embryogenesis [17] and tumor progression in multiple cancers [18–21]. Moreover, it has been reported that the upregulation of SOX4 is related to the neuroendocrine characteristics in small-cell lung cancer [22]. Besides, SOX4 is also involved in the promotion of amino acid and glycerophospholipid metabolism in acute myeloid leukemia [23], and regulation of glycolysis through activation of the AKT pathway in melanoma cells [24]. However, how SOX4 regulates NE trans-differentiation and whether such an effect is achieved by promoting metabolism reprogramming have not been determined. In this study, we provide original evidence for an important role of SOX4 in the regulation of NE trans-differentiation. We demonstrate that SOX4 promotes NE trans-differentiation by activating the SOX4/PCK2 pathway to induce carbohydrate metabolism reprogramming in prostate cancer.

## Methods

### Cell lines and cell culture

The human PCa cell lines BPH-1, LNCaP, C4-2B, PC-3, and DU-145 and human embryonic kidney HEK293T cell lines were obtained from the American Type Culture Collection (ATCC). The LNCaP-shRB1/TP53 cell line has been constructed in our previous work [25]. The BPH-1, 293T, C4-2B, PC-3, and DU-145 cell lines were cultured in Dulbecco’s modified Eagle’s medium (DMEM; Gibco, C11995500BT) supplemented with 10% fetal bovine serum (FBS; Gibco, 10270-106) and 1% penicillin/streptomycin (Beyotime, ST488). The LNCaP and LNCaP-shRB1/TP53 cells were cultured in RPMI-1640 medium (Gibco, C11875500BT) with the same supplements mentioned above. All cells were cultured at 37 °C, in a 5% CO_2_ humid atmosphere. For the experiment of administering enzalutamide, the concentration of enzalutamide used in vitro is 10 µM.

### Plasmids construction

The plasmids containing SOX4-CDS sequence or short hairpin RNA (shRNA) targeting SOX4 were purchased from Genomeditech (Shanghai, China) containing a puromycin-resistant gene (shSOX4 #1: forward 5’- CACCGAGCGACAAGATCCCTTTCATTCTCGAGAATGAAAGGGATCTTGTCGCTTTTTTG -3’, reverse 5’- AAACCAAAAAAGCGACAAGATCCCTTTCATTCTCGAGAATGAAAGGGATCTTGTCGCTC -3’. shSOX4 #2: forward 5’- CACCGTGGGCACATCAAGCGACCCATCTCGAGATGGGTCGCTTGATGTGCCCATTTTTG -3’, reverse 5’-AAACCAAAAATGGGCACATCAAGCGACCCATCTCGAGATGGGTCGCTTGATGTGCCCAC -3’). The shPCK2 plasmid with puromycin-resistance were constructed by inserting the following fragment (forward 5’- CACCGGCACATCCCAACTCTCGATTTCTCGAGAAATCGAGAGTTGGGATGTGCTTTTTG -3’, reverse 5’- AAACCAAAAAGCACATCCCAACTCTCGATTTCTCGAGAAATCGAGAGTTGGGATGTGCC -3’) which was synthesized by Sangon Biotech (Shanghai, China).

### Cell transfection

The lentiviral particle was produced in HEK293T cells after co-transfection with lentiviral plasmid and packaging vectors (psPAX2 and pMD2.G) at a 3:2:1 ratio using Polyetherimide in 10-cm dishes. The lentivirus was harvested 48 h later after replacement of media at 6 h post-transfection. To generate stable transgenic lines, cells were infected at a 50% confluence with the lentiviral particle and 8 µg/mL polybrene (Sigma-Aldrich). The positively transfected cells were selected and enriched by applying puromycin (5-10 µg/mL) in the culture medium for 2 weeks.

### Total RNA extraction and qRT-PCR analysis

Total RNA was extracted from the cells using the FastPure Cell/Tissue Total RNA Isolation Kit, following the manufacturer’s instructions (Vazyme). Subsequently, RNA was reverse-transcribed into cDNA using the HiScript II All-in-one RT SuperMix Perfect qPCR kit (Vazyme, R201-02). qPCR was performed using the qPCR SYBR Green Master Mix (Vazyme, Q711-02). To ensure accuracy and reproducibility, β-actin was used as the internal control gene. All experimental data were obtained in triplicate and analyzed using the 2^−ΔΔCt^ method [26]. The primers are listed in Additional file 2: Table S1.

### Chromatin immunoprecipitation (ChIP)-qPCR assay

The ChIP assay was performed using a SimpleChIP Enzymatic Chromatin IP Kit (CST, 9003s) according to the manufacturer’s instructions. For the assay, 2 × 10^7^ cells were harvested. Briefly, chromatin was crosslinked with nuclear proteins, enzymatically digested with micrococcal nuclease, sonicated, and immunoprecipitated. The normal IgG included in the kit was used as the negative control for IP. The immunoprecipitates were pelleted with agarose beads, purified, and subjected to qPCR using primers specifically targeting the SOX4-binding promoter region. Primer sequences for promoters are listed in Additional file 2: Table S1.

### CCK8 cell viability assay

To determine cell proliferation, cells were seeded on 96-well plates at a density of 2,000 cells per well and cultured in the medium for up to 4 days. Cell proliferation was assessed using the CellTiter96 Aqueous One Solution Cell Proliferation Assay (Biosharp) following the manufacturer’s instructions. The absorbance was measured at 450 nm.

### Colony formation assay

For the colony formation assay, cells were seeded in 12-well plates at a density of 500 cells per well and cultured in the medium for up to 2 weeks. The cells were allowed to grow until visible colonies formed and were then stained with crystal violet (Beyotime).

### Tumor sphere formation assay

Single PCa cells were suspended in a prostate sphere culture medium consisting of DMEM medium supplemented with N_2_ (Gibco), B27 (Gibco), epidermal growth factor (20 ng/mL, PeproTech), and fibroblast growth factor (20 ng/mL, PeproTech). These cells were then seeded in 24-well low-attachment dishes (Corning) at a density of 1,000 cells per well in 500 µL of medium. The culture medium was supplemented every three days until cell spheres formed, which typically occurred after approximately 1 to 2 weeks of culturing. The numbers of spheres were counted using a light microscope (Leica).

### Immunoblotting

Immunoblotting was performed as described in our previous work [27]. Briefly, whole-cell lysates were prepared in radioimmunoprecipitation assay (RIPA) lysis buffer (Millipore) supplemented with a protease inhibitor (MedChemExpress) and phosphatase inhibitor (MedChemExpress). After protein quantification using the Pierce BCA Protein Assay Kit (Thermo Fisher Scientific), leveled protein was separated via SDS-PAGE and transferred to a PVDF membrane (Millipore). The membrane was blocked with TBST containing 5% bovine serum albumin (BSA) at 16-25 °C for 1 h and then incubated with the relevant primary antibodies at 4 °C overnight, followed by probing with a horseradish peroxidase (HRP)-conjugated secondary antibody at 16-25 °C for 1 h. The relevant proteins were visualized using an electrochemiluminescence detection instrument (Bio-Rad) and HRP substrates. The following antibodies were used: SOX4 (Abclonal, A10717), AR (Abcam, ab133273), SYP (Proteintech, 17785-1-AP), NSE (Proteintech, 66150-1-Ig), PCK2 (Abclonal, A21529).

### Hematoxylin-eosin (H&E) and immunohistochemical (IHC) assay

H&E and immunohistochemical staining of paraffin-embedded tissue sections were performed by Runnerbio Biotech. Briefly, the tissues were fixed in 4% paraformaldehyde overnight and embedded in paraffin. Paraffin-embedded tissue sections were dewaxed in xylene for 5 min and successively hydrated in 100%, 95%, 85%, and 70% ethanol. Following the inactivation of endogenous peroxidase with disodium-hydrogen phosphate-2-hydrate, these sections were blocked using 10% donkey serum for 1 h at 16-25 °C for immunohistochemical staining. Next, the sections were incubated with primary antibody (1:100) at 4 °C overnight, washed three times (10 min each time) with PBS, and then incubated with horseradish peroxidase-conjugated secondary antibody (Vector Laboratories, Burlingame, CA, USA) for 1 h at 16-25 °C. Finally, after washing three times with PBS, the sections were visualized with diaminobenzidine (DAB) staining (Sangon Biotech) and hematoxylin counterstaining (Beyotime). Images were acquired using a microscope (Leica). IHC analysis of cell line-derived xenograft and clinical tumor samples was performed using antibodies against SOX4 (Abclonal, A10717), AR (Abcam, ab133273), NSE (Proteintech, 66150-1-Ig), SYP (Proteintech, 17785-1-AP), Ki67 (Abcam, ab15580), or PCK2 (Abclonal, A21529), following a published protocol. For statistics, 3-5 fields per section were randomly selected, and each field was scored by the product of staining intensity (1, 2, 3, 4) and staining area (25%, 50%, 75%, 100%). The mean value of the score was used to compare the difference between the CRPC-Ad and NEPC groups using the Mann-Whitney test.

### Transwell assay

In the assessment of cell migration via the transwell assay, the procedure commenced with the inoculation of the upper compartment with a suspension of 5 × 10^4^ DU-145, PC-3 or C4-2B cells, using 150 µL of DMEM Basic Medium (Gibco) per well. Simultaneously, the bottom compartment was filled with 600 µL of DMEM, enriched with 10% FBS. This system was then incubated for 24 h. The cells that had migrated to the lower membrane surface were fixed with a 4% paraformaldehyde solution for 15 min. Afterward, the cells were stained with crystal violet (Beyotime) at room temperature for approximately 20 min, then photographed using a microscope (Leica) and counted using ImageJ (version 1.53k).

### Luciferase assay

To determine the effect of SOX4 on PCK2 promoter-luciferase activity, 293T cells were transfected with WT or mutant PCK2 promoter-luc constructs along with pRL-TK. After transfection, cells were harvested, and cell lysates were prepared. The relative luciferase activity was measured using the Dual-Luciferase Reporter Assay (Promega, Germany) according to the manufacturer’s instructions. The primer sequences for cloning PCK2 promoter were: forward 5’- GCGTGCTAGCCCGGGCTCGAGCATGCACAGTGTGGGGAGTGG -3’ and reverse 5’-CAGTACCGGAATGCCAAGCTTGGCGCGGGGGCGGAACCT -3’, the primer sequences for cloning PCK2 mutant promoter were: forward 5’- CTGTAACTGAAATCAGGAACAAGCCTACAGCCATCTCCCTGCTCTGCT -3’ and reverse 5’- AGCAGAGCAGGGAGATGGCTGTAGGCTTGTTCCTGATTTCAGTTACAG -3’.

### Metabolites measurement

Cells were seeded in 12-well plates at a density of 10,000 cells per well and were cultured in the medium of different glucose concentrations for up to 48 h. The cell culture supernatant was collected for the glucose and lactate quantification using Glucose kit and Lactate kit (Cedex Bio) respectively, using a Roche Cedex Bio Analyzer. The cell pellet was used for pyruvic acid measurement, which was performed according to the manufacturer’s instructions (Nanjing Jiancheng, A081-1-1).

### Tumor xenograft experiment

All procedures involving animals were performed in strict alignment with the guidelines sanctioned by the Ren Ji Hospital Institutional Animal Care and Use Committee. Euthanasia was performed before any specimen exhibited a body weight reduction exceeding 20%. Housing for murine subjects was provided in a controlled, pathogen-free environment within the facilities of Ren Ji Hospital (approval number: RA-2022-191). In order to mimic a CRPC-like tumor environment and to avoid potential interference of androgens on tumor growth [28–30], the nude mice were castrated 14 days before the injection of tumor cells. Meanwhile, daily intraperitoneal administrations of enzalutamide (Med Chem Express) at a concentration of 10 mg per kilogram of body weight were given. For in vivo investigations, 5 million cells were resuspended in 100 µl of a 50% Matrigel solution and injected subcutaneously into the right flank of nude mice. After euthanasia, tumor specimens were collected for imaging and mass measurement to evaluate the experimental outcomes.

### Clinical samples

Experimental procedures involving human subjects were executed in accordance with ethical standards outlined in the World Medical Association’s Declaration of Helsinki and conformed to both local and international regulatory requirements. The analysis of human samples was approved by the Committee for Ethics of Ren Ji Hospital (approval number: KY2019-081), and written informed consent was obtained from all patients before their participation. For IHC assay, PCa tissue samples were obtained from the Urology Department of Ren Ji Hospital. Comprehensive clinical profiles of the subjects enrolled in the study are documented in Additional file 2: Table S2.

### Bioinformatics analysis

The identification of gene expression alterations between experimental and control cohorts within the Gene Expression Omnibus (GEO) database and Cbioportal database. Detailed information about the analysis method of each dataset used was shown in Additional file 2: Table S3. Briefly, we analyzed the genes’ expression levels using R with DESeq2 or limma [31] package according to the type of raw data. Pearson’s correlation analyses were conducted to generate correlation coefficients. Besides, online websites inculding CANCERTOOL (http://web.bioinformatics.cicbiogu-ne.es/CANCERTOOL/index.html) and PanCanSurvPlot (https://smuonco.shinyapp-s.io/PanCanSurvPlot/) were used to evaluate the correlations between genes expression and patients’ survival. HIPLOT (https://hiplot.com.cn/home/index.html) was used to draw the heatmap.

### RNA-seq, ATAC-seq and untargeted metabolomics

The mRNA was converted into barcoded cDNA fragments by reverse transcription, utilizing an oligo-dT primer appended with an adapter. These barcoded cDNA libraries were sequenced on the Illumina NovaSeq 6000 (Illumina). Following the quality control evaluation, the RNA-seq reads were aligned to the reference genome (GRCh38/hg38) using HISAT2. StringTie facilitated the assembly and quantification of transcript abundances. Differential gene expression analysis was conducted on the normalized data with DESeq2. Each cell line was represented by three replicates in the study.

For the ATAC-seq assay, 50,000 cells were centrifuged at 500 g for 5 min at 4 °C, and the supernatant discarded. The cells were then washed once with cold PBS. Following this, they were centrifuged again at 500 g for 5 min at 4 °C, and the supernatant was removed. Subsequently, the cells were resuspended in a cold lysis buffer. The cells were centrifuged once more at 500 g for 10 min at 4 °C, and the supernatant discarded. The transposition reaction mixture was prepared using Tn5 transposase. The cell nuclear content was added to this mixture, and the DNA was purified after incubating at 37 °C for 30 min. The PCR system was set up with the purified DNA, and PCR amplification was performed. The final DNA libraries were sequenced on an Illumina platform after purification. The integrative genome browser (IGV) program was utilized for peak visualization. Each cell line was represented by two replicates in this experiment.

For metabolite analysis, cells were collected by cell scrapers after being washed with PBS 3 times. Each sample contained more than 1 × 10^7^ cells. The cells were centrifuged at 1000 rpm for 5 min at 4 °C. The supernatant was discarded and the sample was stored at -80 °C. The samples were used for LC-MS detection and analysis by PANOMIX Biomedical Technology (Suzhou, China). The LC analysis was performed on a Vanquish UHPLC System (Thermo Fisher Scientific, USA). Mass spectrometric detection of metabolites was performed on Orbitrap Exploris 120 (Thermo Fisher Scientific, USA) with an ESI ion source.

### Statistical analysis

Experimental data were presented as mean and standard deviation (SD), derived from a minimum of three independent assays unless specified otherwise. Unpaired two-tailed Student’s t-test was used to ascertain statistical discrepancies between binary comparisons. For assessments involving more than two variables, one-way Analysis of Variance (ANOVA) was utilized. For ordinal data, Mann-Whitney U test was utilized. Statistical significance was set at a *p*-value threshold of less than 0.05.

## Results

### The expression of SOX4 is upregulated in NEPC

**Fig. 1:**
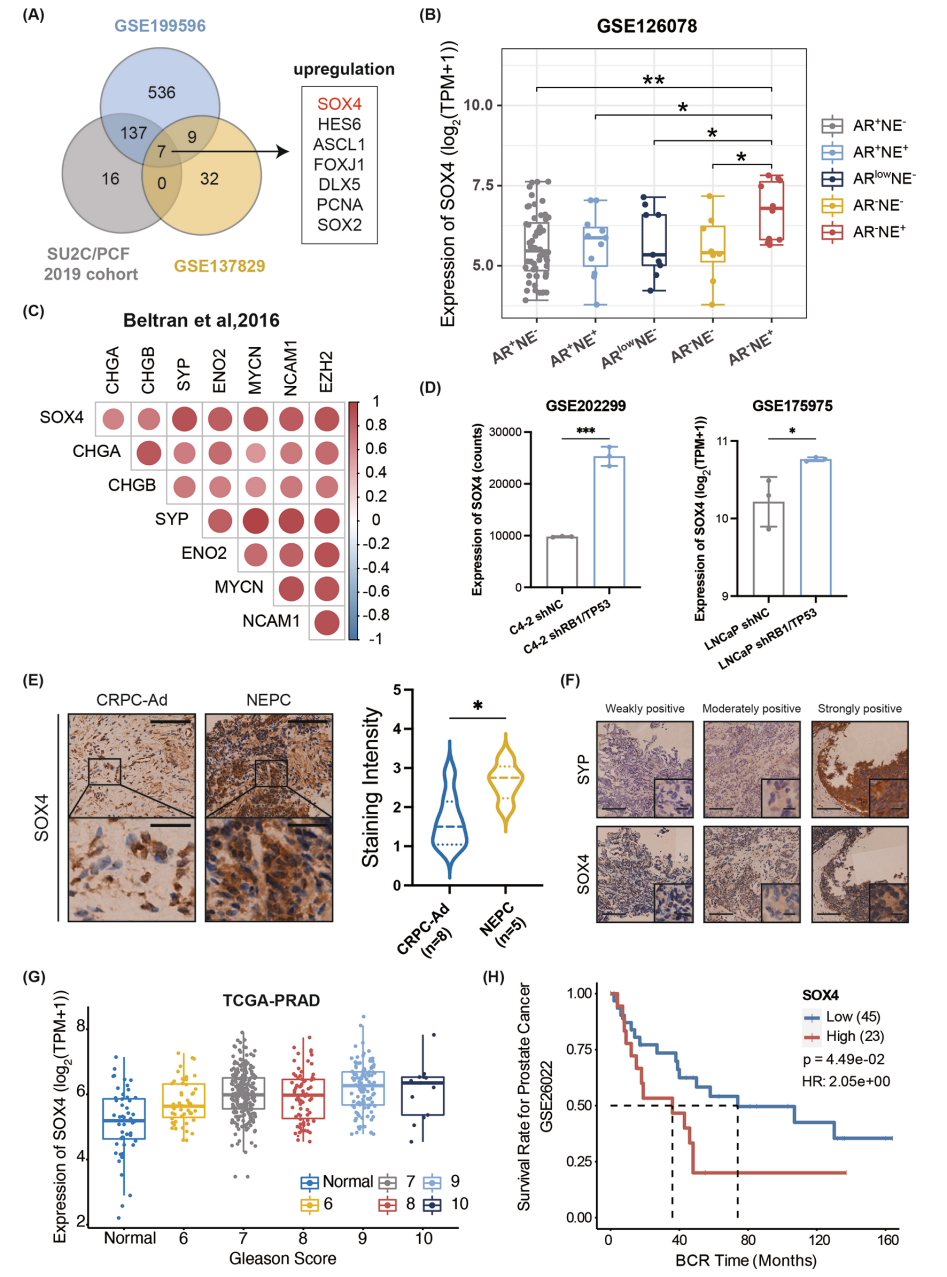
SOX4 expression is mostly upregulated in NEPC. **(A)** Intersection of significantly upregulated transcription factors in NEPC vs. CRPC samples. **(B)** Comparison of SOX4 mRNA levels in different phenotypes of PCa using data from GSE126078. **(C)** Pearson’s correlation analysis of SOX4 vs. NE signature genes from Beltran 2016 cohort. **(D)** Comparison of SOX4 mRNA levels in prostate cancer cell lines using data from GSE202299 and GSE175975. **(E)** Representative IHC staining of SOX4 in clinical tumor sections of CRPC-Ad (*n* = 8) and NEPC (*n* = 5). Upper panel scale bar: 100 μm, lower panel scale bar: 25 μm. **(F)** Representative IHC staining of SYP and SOX4 using continuous tissue sections. Whole panel scale bar: 50 μm, small panel scale bar: 20 μm. **(G)** SOX4 expression in patients with different Gleason scores was analyzed using data from the TCGA database. **(H)** BCR time among PCa patients with high or low expression levels of SOX4. Two-tailed Student’s t-test was used for statistical analysis: ns, not significant; *, *p* < 0.05; **, *p* < 0.01; ***, *p* < 0.001; ****, *p* < 0.0001

To identify important candidate genes involved in the regulation of NE trans-differentiation, we conducted bioinformatics assays using public datasets. We screened expression changes of transcription factors from 2 independent datasets: SU2C/PCF 2019 cohort (clinical samples directly collected from NEPC and CRPC patients) and GSE199596 (patient-derived xenografts with AR or NE characteristics classified by Coleman et al.). Besides, we also summarized a list of TFs involving in NE-related meta programs [32] which were derived from a single-cell analysis of clinical diagnosed CRPC and NEPC patients (GSE137829). Next, we analyzed the intersection of all the candidate genes and sorted out a total of 7 TFs that exhibited a significant upregulation in NEPC samples (Fig. 1A, Additional file 1: Fig. S1A). Furthermore, in dataset GSE126078, which was composed of clinical samples with variant AR and NE marker profiling, SOX4 expression was observed to be the most increased in AR^−^NE^+^ samples, representing the common represented NEPC subtype [33] (Fig. 1B). Moreover, we analyzed the expression relationship between SOX4 and NE marker genes in the Beltran 2016 cohort and found a positive expression correlation of SOX4 with well-known NE marker genes such as SYP, CHGA, CHGB, and ENO2 (Fig. 1C). In addition, we also examined the expression of SOX4 using RNA-seq data derived from the RB1/TP53 double knockdown LNCaP (GSE175975) or C4-2 cells (GSE202299), which serve as well-known NEPC-like cell lines. We confirmed the upregulation of SOX4 in both datasets as compared to their respective controls (Fig. 1D). Similarly, the expression of both SOX4 and ENO2 was shown to be increased along with long period androgen deprivation treatment in the LNCaP cell line. (Additional file 1: Fig. S1B). Therefore, these bioinformatic assay results indicated that the elevated expression of SOX4 was involved in the regulation of NE trans-differentiation.

For further verification, we performed the IHC assay on tumor sections from clinical patients of CRPC-Ad or NEPC and analyzed the staining intensity differences of SOX4. Consistent with the results from bioinformatic assays, we observed the elevated SOX4 expression in NEPC samples (Fig. 1E). We also used continuous tissue sections from the same patient to perform IHC staining of SOX4 and SYP, one of the NE marker genes, and confirmed the high expression of SOX4 in high-SYP-expressing fields (Fig. 1F). In addition, we examined the expression of SOX4 in a PCa clinical tissue chip with negative, weakly positive, moderately positive or strongly positive expression of SYP (Additional file 1: Fig. S1C). By analyzing data from the TCGA database, we found that SOX4 expression was continuously upregulated from Gleason score 6 to 10 (Fig. 1G). Moreover, we found that patients with higher SOX4 expression levels had a shorter biochemical recurrence time (BCR) in the GSE26022 dataset (Fig. 1H). Taken together, these findings indicated that expression of SOX4 is positively correlated with NE trans-differentiation.

### SOX4 promotes the malignant phenotypes and the NE trans-differentiation in PCa cells in vitro

**Fig. 2:**
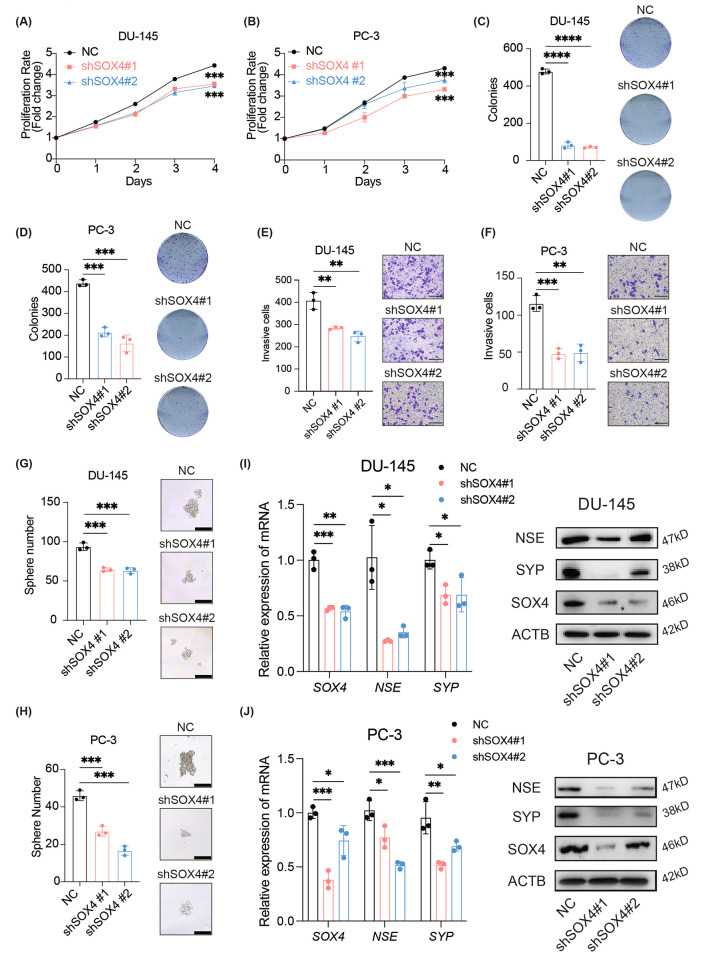
SOX4 promotes the malignant phenotypes and the NE trans-differentiation in PCa cells in vitro. **(A)** Cell proliferation assay in SOX4-knockdown DU-145 cells. **(B)** Cell proliferation assay in SOX4-knockdown PC-3 cells. **(C)** Representative images and quantification of colony numbers in SOX4-knockdown DU-145 cells. **(D)** Representative images and quantification of colony numbers in SOX4-knockdown PC-3 cells. **(E)** Representative images and quantification of invasive cells after SOX4 knockdown in DU-145 cells. Scale Bar: 200 μm. **(F)** Representative images and quantification of invasive cells after SOX4 knockdown in PC-3 cells. Scale Bar: 200 μm. **(G)** Representative images and quantification of tumor sphere formation after SOX4 knockdown in DU-145 cells. Scale Bar: 50 μm. **(H)** Representative images and quantification of tumor sphere formation after SOX4 knockdown in PC-3 cells. Scale Bar: 50 μm. **(I)** mRNA and protein expression of SOX4, NSE and SYP in SOX4-knockdown DU-145 cells. **(J)** mRNA and protein expression of SOX4, NSE, and SYP in SOX4-knockdown PC-3 cells. All experiments were performed in triplicate and were repeated three times. Two-tailed Student’s t-test was used for statistical analysis: *, *p* < 0.05; **, *p* < 0.01; ***, *p* < 0.001; ****, *p* < 0.0001. Data are presented as means ± SD, *n* = 3

Next, we further investigated whether elevated SOX4 expression could promote the NE trans-differentiation and other malignant phenotypes such as cell proliferation and migration in PCa. To this end, we examined endogenous SOX4 expression levels in several PCa cell lines and found that while DU-145 and PC-3 cells expressed high levels of SOX4, C4-2B cells expressed low levels of SOX4 (Additional file 1: Fig. S2A). It has been previously reported that DU-145 and PC-3 cells are androgen-independent cell lines [34], and C4-2B cells are androgen receptor-positive but androgen-independent [35]. All three cell lines possess a tendency to develop NE characteristics [36, 37]. Thus, we employed SOX4-high-expressing DU-145 and PC-3 cells to construct SOX4-knockdown subclone cell lines and selected SOX4-low-expressing C4-2B cells to construct SOX4-overexpressing subclone cell lines. We observed that the knockdown of SOX4 significantly inhibited cell proliferation (Fig. 2A and B) and exhibited a repressive effect on colony formation (Fig. 2C and D). SOX4 knockdown also downregulated the ability of cell migration (Fig. 2E and F) and sphere formation (Fig. 2G and H) at the same time. Notably, SOX4 knockdown reduced the expression of NE markers SYP and NSE in DU-145 and PC-3 cells compared to controls at both the protein and mRNA levels (Fig. 2I and J). In addition, knockdown of SOX4 in the NEPC cell line LNCaP-shRB1/TP53 [38] also resulted in the downregulation of NE marker gene expression, along with the attenuation of its resistance to ENZ (Additional file 1: Fig. S2B-S2C).

**Fig. 3:**
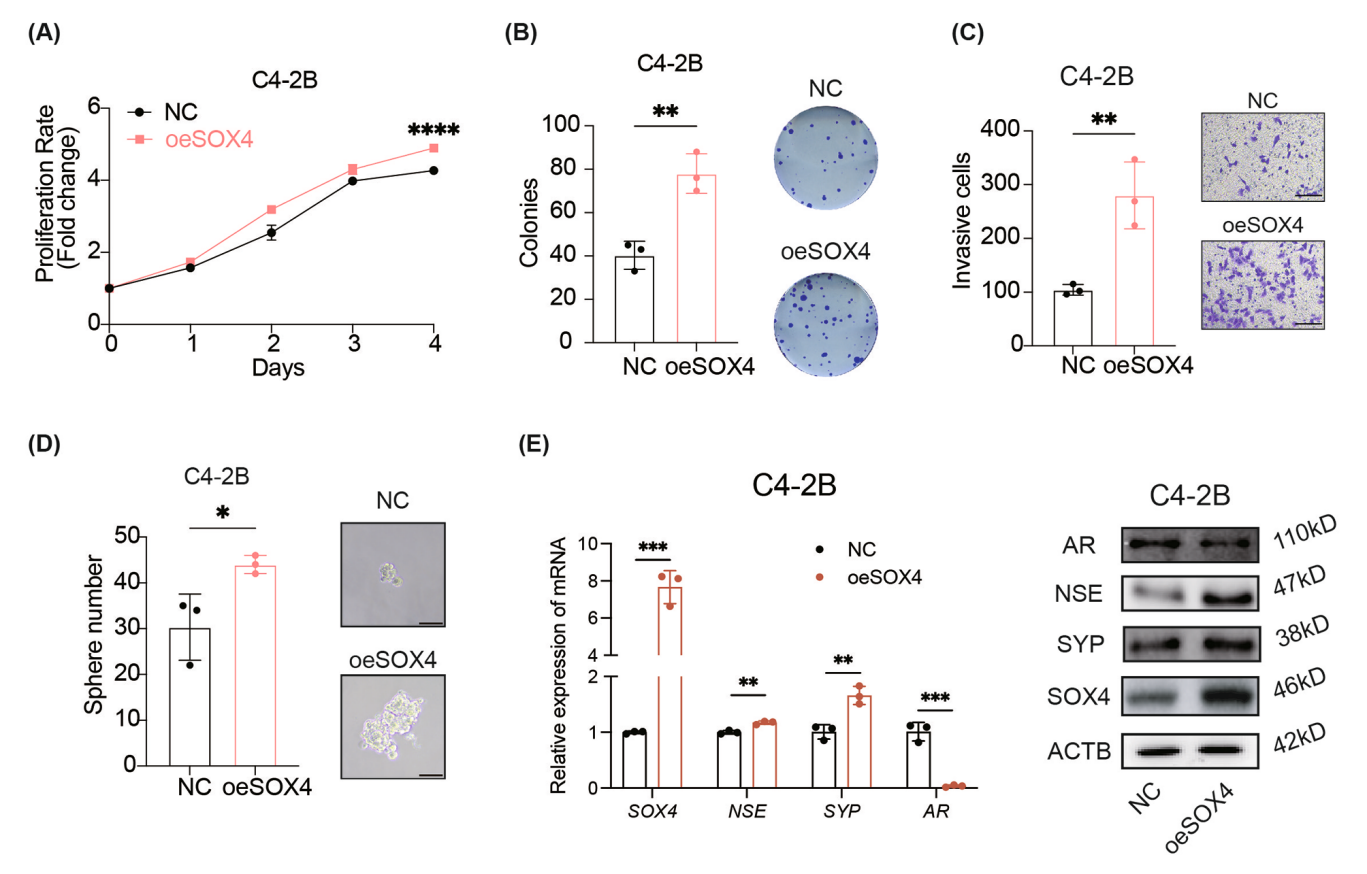
SOX4 promotes the malignant phenotypes and the NE trans-differentiation in PCa cells in vitro. **(A)** Cell proliferation assay in SOX4-overexpressed C4-2B cells. **(B)** Representative images and quantification of colony numbers in SOX4-overexpressed C4-2B cells. **(C)** Representative images and quantification of invasive cells after SOX4-overexpressed in C4-2B cells. Scale Bar: 200 μm. **(D)** Representative images and quantification of tumor sphere formation after SOX4-overexpressed in C4-2B cells. Scale Bar: 50 μm. **(E)** mRNA and protein expression of SOX4, NSE, SYP and AR in SOX4-overexpressed C4-2B cells. All experiments were performed in triplicate and were repeated three times. Two-tailed Student’s t-test was used for statistical analysis: ns, not significant; *, *p* < 0.05; **, *p* < 0.01; ***, *p* < 0.001; ****, *p* < 0.0001. Data are presented as means ± SD, *n* = 3

Consistent with the above findings, overexpression of SOX4 accelerated the proliferation and colony formation of C4-2B cells compared to the control (Fig. 3A and B). Besides, SOX4 overexpression enhanced the migration ability of C4-2B cells (Fig. 3C). The tumor sphere formation ability was also upregulated in C4-2B-oeSOX4 cells (Fig. 3D). Also, upregulation of SOX4 enhanced the expression of SYP and NSE, and downregulated AR expression at both mRNA and protein levels (Fig. 3E and Additional file 1: Fig. S3A-S3C). We also investigated the expression level of SOX4-related genes by IHC assay using sections of tumor samples from clinical patients. Consistent with the observations in cell lines, the elevated expression of SOX4 regulated-genes SYP and NSE was verified while AR remained negative in NEPC samples compared to that from CRPC (Additional file 1: Fig. S3D). Collectively, these results indicate that SOX4 is essential for promoting NE trans-differentiation.

### SOX4 regulates carbohydrate metabolism reprogramming in prostate cancer

**Fig. 4:**
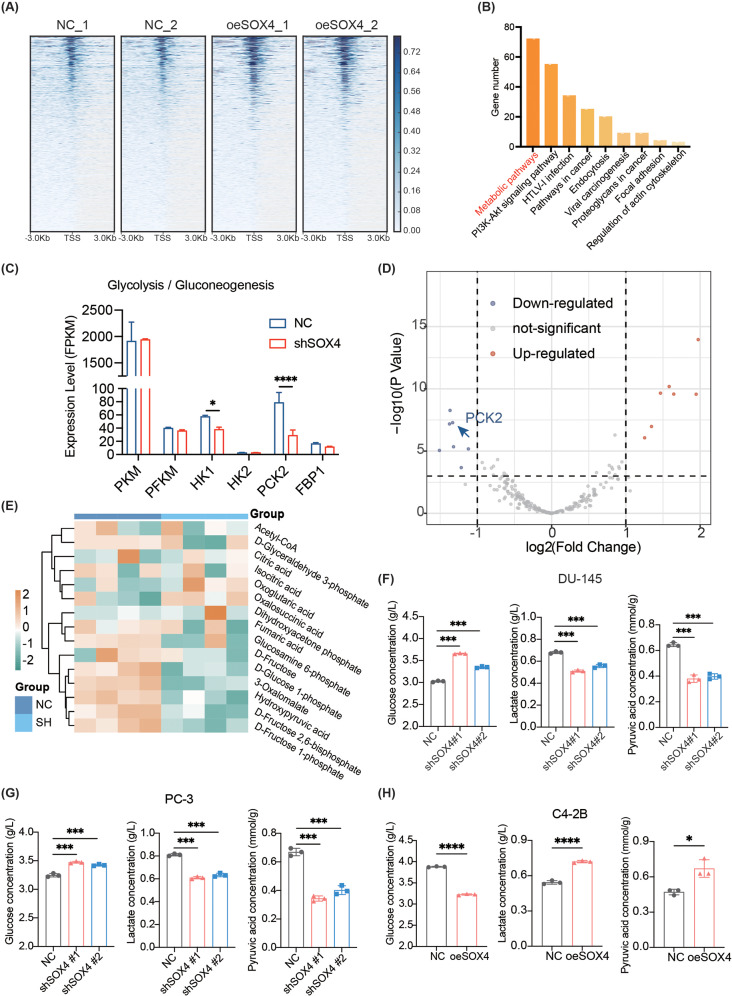
SOX4 regulates carbohydrate metabolism reprogramming in PCa. **(A)** Heatmap for the density distribution of sequencing reads in the 3 kb interval upstream and downstream of the TSS of each gene. **(B)** KEGG enrichment analysis of ATAC-seq. **(C)** Expression of key enzymes related to glycolysis/gluconeogenesis in RNA-seq. **(D)** Volcano plot of differential analysis on genes related to carbohydrate metabolism using RNA-seq data of DU-145-shSOX4 cells vs. shNC cells. **(E)** Heatmap for the relative content of metabolites related to the process of glycolysis/gluconeogenesis and TCA cycle. **(F)** The concentration changes of glycolysis-related metabolites in DU-145-shSOX4 cells vs. shNC cells. **(G)** The concentration changes of glycolysis-related metabolites in PC-3-shSOX4 cells vs. shNC cells. **(H)** The concentration changes of glycolysis-related metabolites in C4-2B-oeSOX4 cells vs. oeNC cells. All experiments were performed in triplicate and were repeated three times. Two-tailed Student’s t-test was used for statistical analysis: *, *p* < 0.05; **, *p* < 0.01; ***, *p* < 0.001; ****, *p* < 0.0001. Data are presented as means ± SD, *n* = 3

Next, we attempted to explore the underlying mechanism by which SOX4 promotes NE trans-differentiation in PCa cells. Since chromatin accessibility is closely related to transcriptional regulation, we performed ATAC-seq to investigate whether SOX4 could promote NE trans-differentiation by improving cell plasticity. As shown in Fig. 4A, chromatin accessibility was increased after SOX4 overexpression compared to that in control cells. Furthermore, we carried out KEGG enrichment analysis using our ATAC-seq data and we found that more than seventy genes were enriched in the metabolic pathways, which indicated that SOX4 might affect metabolic reprogramming (Fig. 4B).

Considering that the shift from oxidative phosphorylation to aerobic glycolysis plays an important role in tumor metabolism during the progression from CRPC to NEPC [39], we focused on investigating whether SOX4 could regulate the metabolism reprogramming, especially carbohydrate metabolism reprogramming, to promote NE trans-differentiation in tumor cells. To this end, we performed RNA-seq on DU-145-shSOX4 versus control cells to screen potential candidate genes as downstream effectors. We found that among the well-known key enzymes related to glycolysis/gluconeogenesis and lipid metabolism, only the expression of HK1 and PCK2 was significantly downregulated after SOX4 knockdown, and that between the two genes, PCK2 expression exhibited a much more obvious reduction (Fig. 4C). However, lipid metabolism-related genes exhibited no significant change after SOX4 knockdown (Additional file 1: Fig. S4A), indicating a selective involvement of PCK2-mediated glycolysis/gluconeogenesis than lipid metabolism. Furthermore, differential gene expression analysis (considering significance at *P* < 0.001 and |log2FC| > 1) on genes related to carbohydrate metabolism also indicated a significant decrease in PCK2 expression in SOX4-knockdown cells compared to control cells (Additional file 2: Table S4), suggesting PCK2 as a promising downstream effector of SOX4 on regulating carbohydrate metabolism reprogramming in PCa cells (Fig. 4D). As additional support for our finding, data from the non-targeted metabolomics sequencing also revealed a significant decrease in the content of glycolysis/gluconeogenesis-related metabolites and an increase on the content of tricarboxylic acid cycle-related metabolites after knockdown of SOX4 (Fig. 4E and Additional file 2: Table S5). Taken together, our results indicate that elevated expression of SOX4 appears to promote carbohydrate metabolism reprogramming for NE trans-differentiation via PCK2 as its downstream effector.

Because the enhancement of aerobic glycolysis is a major characteristic of carbohydrate metabolism reprogramming in PCa [40], we further investigated whether SOX4 is involved in the regulation of aerobic glycolysis. It has been reported that the intake of glucose and the production of both pyruvic acid and lactate decrease during the process of aerobic glycolysis [41]. Thus, we examined the content of glucose and lactate in cell culture supernatants and the intracellular pyruvic acid content in different cell lines respectively. We found that in DU-145-shSOX4 or PC-3-shSOX4 cells, the glucose content was increased in the supernatant as compared to the control, reflecting an attenuated intake of glucose in tumor cells after SOX4 knockdown. Consistently, the lactate content in the supernatant and the intracellular pyruvic acid content were decreased, indicating the repression of the production of these two compounds (Fig. 4F and G). As expected, an opposite result was observed in C4-2B-oeSOX4 cells in which overexpression of SOX4 promoted aerobic glycolysis (Fig. 4H). We also measured metabolite concentrations in LNCaP-shRB1/TP53 cells and their control cells. Consistent with the former results, while an enhancement of aerobic glycolysis in LNCaP-shRB1/TP53 was observed, knockdown of SOX4 in the cells could reverse the process (Additional file 1: Fig. S4B). Thus, considering these findings together, we revealed that SOX4 induces carbohydrate metabolism reprogramming mainly by promoting aerobic glycolysis and that this process might be mediated by SOX4/PCK2 signaling.

### SOX4 enhances aerobic glycolysis to promote NE trans-differentiation via activation of PCK2

**Fig. 5:**
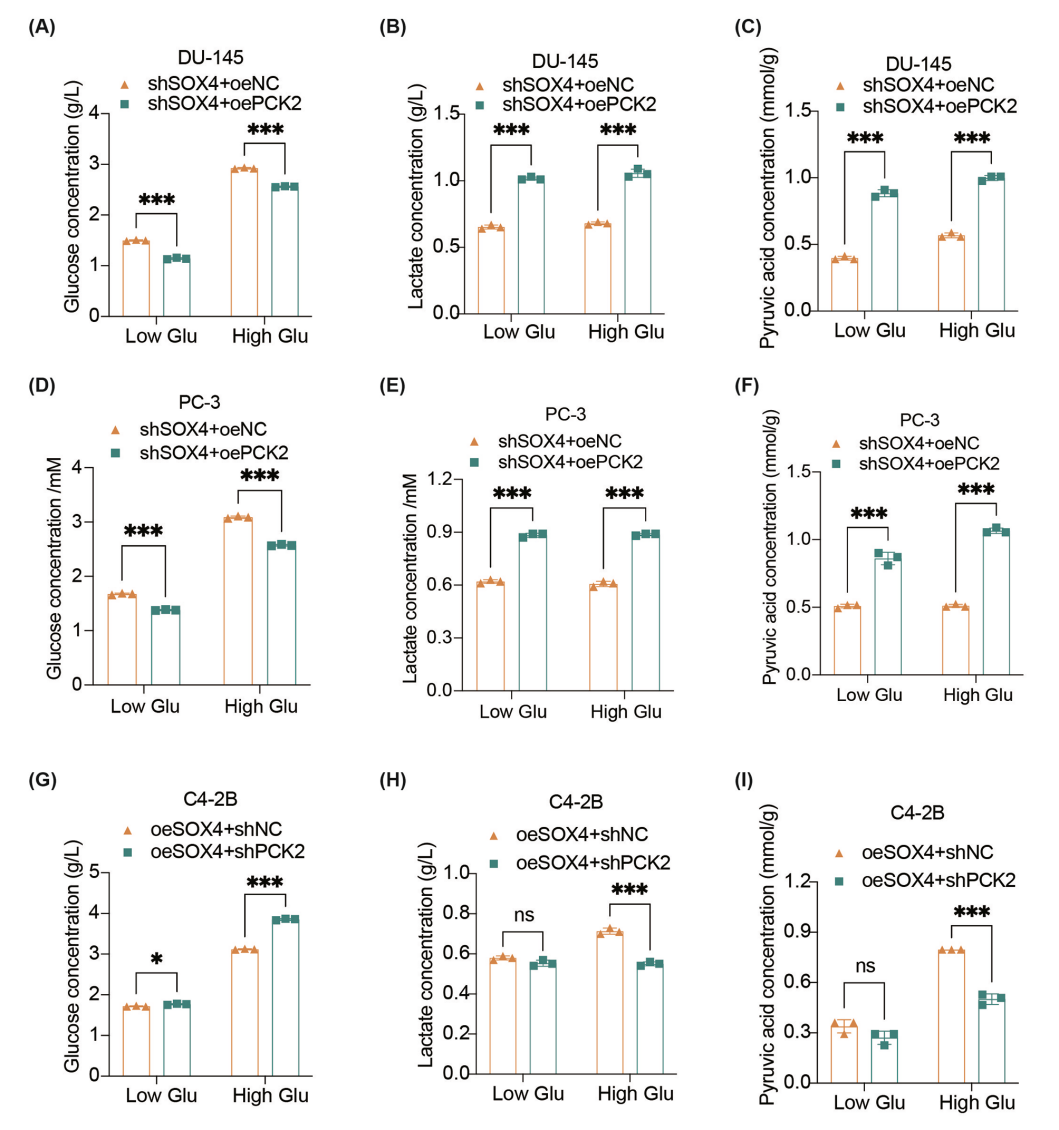
SOX4 enhances aerobic glycolysis to promote NE trans-differentiation via activation of PCK2. **(A-C)** The concentration of glucose **(A)** and lactic acid **(B)** in the culture supernatant of DU-145 cells or pyruvic acid **(C)** intracellular after knockdown of SOX4 plus either PCK2 overexpression or the relevant control cultured under low or high glucose conditions. **(D-F)** The concentration of glucose **(D)** and lactic acid **(E)** in the culture supernatant of PC-3 cells or pyruvic acid **(F)** intracellular after knockdown of SOX4 plus either PCK2 overexpression or the relevant control cultured under low or high glucose conditions. **(G-I)** The concentration of glucose **(G)** and lactic acid **(H)** in the culture supernatant of C4-2B cells or pyruvic acid **(I)** intracellular after overexpression of SOX4 plus either PCK2 knockdown or the relevant control cultured under low or high glucose conditions. All experiments were performed in triplicate and were repeated three times. Two-tailed Student’s t-test was used for statistical analysis: ns, not significant; *, *p* < 0.05; **, *p* < 0.01; ***, *p* < 0.001. Data are presented as means ± SD, *n* = 3

To confirm whether PCK2 was the main downstream effector of SOX4 to promote aerobic glycolysis, we first examined expression levels of PCK2 in the SOX4 knockdown or overexpression subclone cell lines at both mRNA and protein levels. We found that PCK2 was significantly decreased after SOX4 knockdown but increased after SOX4 overexpression (Additional file 1: Fig. S5A-C). In rescue assays, we knocked down PCK2 in C4-2B-oeSOX4 cells. Conversely, PCK2 was overexpressed after SOX4 knockdown in DU-145 or PC-3 cells respectively (Additional file 1: Fig. S5D). For further confirmation of SOX4/PCK2-mediated enhancement of aerobic glycolysis, we cultured cells in low or high glucose media respectively, and repeated the above experiments to measure the content of metabolites. We found that after overexpression of PCK2, with either low glucose or high glucose treatment, the content of glucose in the supernatant was again decreased even under SOX4 knockdown in both DU-145 and PC-3 cells (Fig. 5A and D). Meanwhile, the production of lactate and pyruvic acid was increased under either low or high glucose culture, which indicated that overexpression of PCK2 could effectively reverse the phenotypes obtained by SOX4 knockdown (Fig. 5B-C and E-F). Similarly, PCK2 knockdown in C4-2B-oeSOX4 cells resulted in an increased glucose content in the supernatant and a decreased production of both lactate and pyruvic acid in cultures with high glucose (Fig. 5G-I). These results indicated that PCK2 could work as a downstream effector of SOX4 to enhance the process of aerobic glycolysis.

**Fig. 6:**
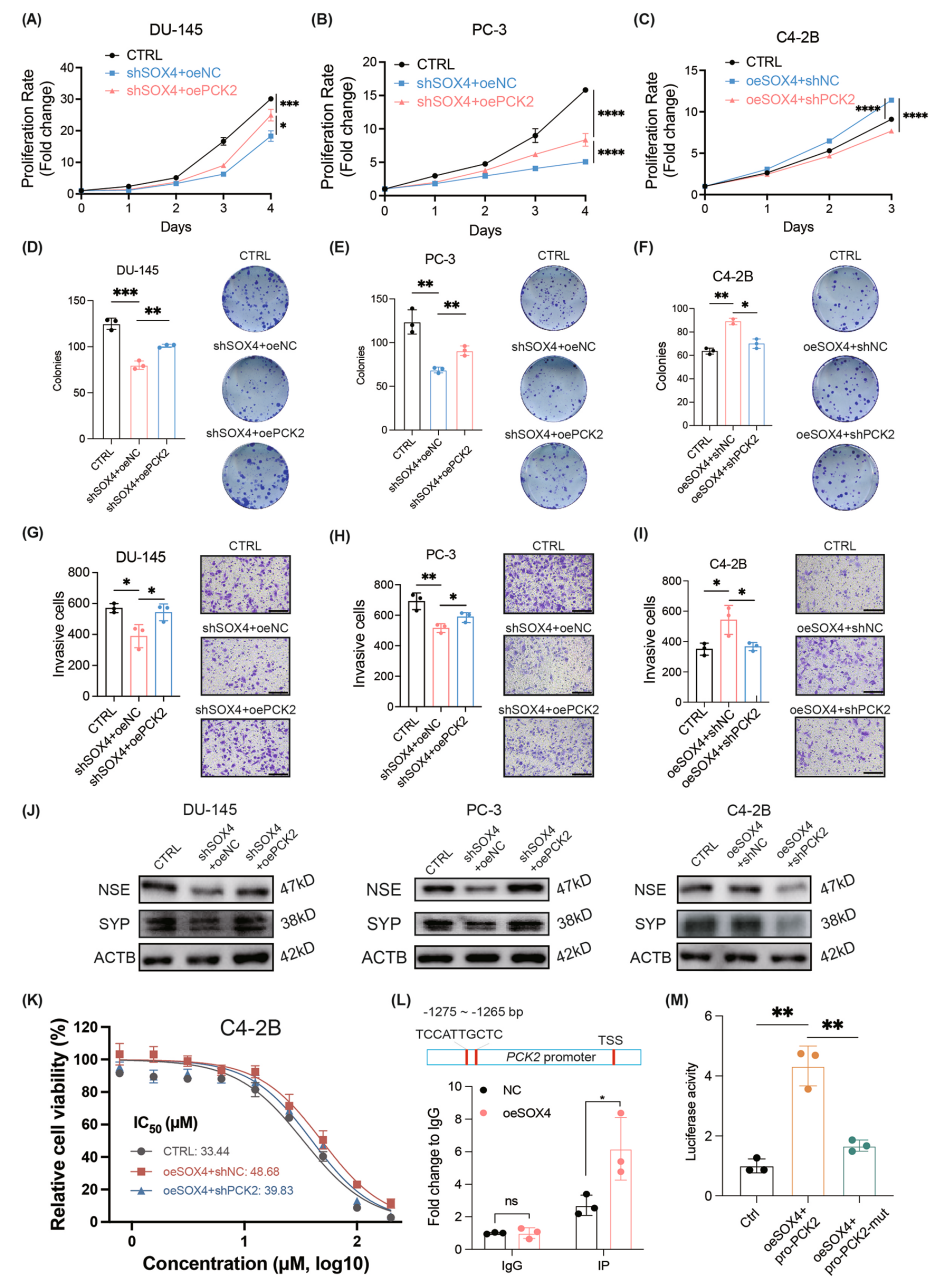
SOX4 promotes malignant phenotypes in PCa via PCK2. **(A)** Cell proliferation assay in SOX4-knockdown DU-145 cells with or without PCK2 overexpression. **(B)** Cell proliferation assay in SOX4-knockdown PC-3 cells with or without PCK2 overexpression. **(C)** Cell proliferation assay in SOX4-overexpressed C4-2B cells with or without PCK2 knockdown. **(D)** Representative images and quantification of colonies in SOX4-knockdown DU-145 cells with or without PCK2 overexpression. **(E)** Representative images and quantification of colonies in SOX4-knockdown PC-3 cells with or without PCK2 overexpression. **(F)** Representative images and quantification of colonies in SOX4-overexpressed C4-2B cells with or without PCK2 knockdown. **(G)** Representative images and quantification of invasive cells after SOX4 knockdown in DU-145 cells with or without PCK2 overexpression. Scale Bar: 200 μm. **(H)** Representative images and quantification of invasive cells after SOX4 knockdown in PC-3 cells with or without PCK2 overexpression. Scale Bar: 200 μm. **(I)** Representative images and quantification of invasive cells after SOX4 overexpression in C4-2B cells with or without PCK2 knockdown. Scale Bar: 200 μm. **(J)** Protein expression of NSE, SYP and ACTB in SOX4-knockdown cells with or without PCK2 overexpression and in SOX4-overexpressed cells with or without PCK2 knockdown. **(K)** IC_50_ curves of enzalutamide resistance in C4-2B-oeSOX4 cells with or without PCK2 knockdown. **(L)** ChIP-qPCR assay of SOX4 binding on the promoter of PCK2 in C4-2B cells. **(M)** Verification of the SOX4 binding site on the promoter region of PCK2 through luciferase assay. All experiments were performed in triplicate and were repeated three times. Two-tailed Student’s t-test was used for statistical analysis: ns, not significant; *, *p* < 0.05; **, *p* < 0.01; ***, *p* < 0.001; ****, *p* < 0.0001. Data are presented as means ± SD, *n* = 3

Additionally, by cell proliferation assay, we observed that overexpression of PCK2 in DU-145 and PC-3 cells promoted cell proliferation, which was inhibited by SOX4 reduction (Fig. 6A and B). Conversely, when PCK2 was knocked down in C4-2B cells, the cell proliferation was inhibited even through SOX4 was overexpressed (Fig. 6C). Moreover, as rescue assays, overexpression of PCK2 improved, and knockdown of PCK2 attenuated, cell colony formation (Fig. 6D-F) and cell migration (Fig. 6G-I). Consistent with this observation on phenotypes, the expression of NE markers NSE and SYP was downregulated after SOX4 knockdown but upregulated following the overexpression of PCK2. Also, PCK2 knockdown downregulated the expression of these genes under the condition of SOX4 overexpression (Fig. 6J). Besides these findings, the expression relationship assay indicated a positive correlation between PCK2 expression and CHGA, NSE or SYP expression (Additional file 1: Fig. S5E). Meanwhile, we also evaluated the resistance to ENZ treatment in C4-2B cells and found that the overexpression of SOX4 significantly enhanced the IC_50_ value, while PCK2 knockdown weakened the ability (Fig. 6K). Thus, all these data indicated that SOX4 promotes the NE trans-differentiation via the SOX4/PCK2 axis.

To further explore the detailed regulatory mechanism of SOX4 on PCK2 expression, we used JASPAR to predict whether SOX4 could promote PCK2 expression at the transcriptional level. We found that there was a potential SOX4 binding site (TCCATTGCTC) harbored at the promoter region of PCK2 (-1275 ~ -1265 bp before the transcription start site). By ChIP-qPCR assay, the enrichment of the related DNA fragment was observed in C4-2B-oeSOX4 cells compared to the control (Fig. 6L). Moreover, the result from the dual luciferase reporter assay also indicated that SOX4 could recognize and bind to the promoter of PCK2 to directly activate its transcription (Fig. 6M). Collectively, all the above results support the notion that apart from directly transcriptional regulated NE markers and AR expression, SOX4 also enhances aerobic glycolysis to promote NE trans-differentiation via activation of PCK2.

### Knockdown of SOX4 inhibits tumor growth and NE markers expression in vivo

**Fig. 7:**
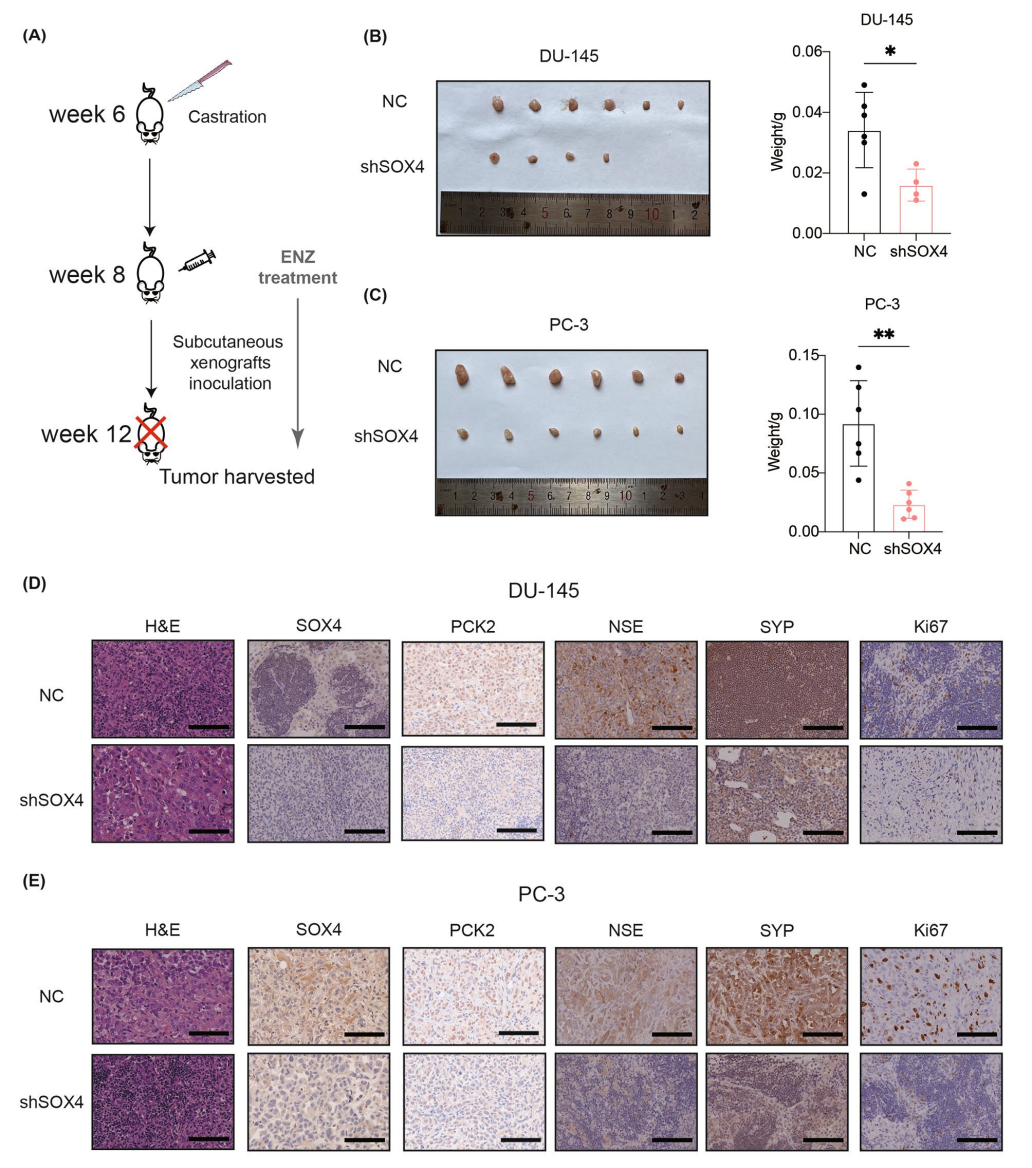
SOX4 inhibits PCa tumor growth in vivo. **(A)** Schematic flowchart of castration nude mouse subcutaneous tumor model establishment. **(B)** Anatomical tumor images and of tumor weight statistics of DU-145-shSOX4 or the control cell-inoculated xenografts. **(C)** Anatomical tumor images and of tumor weight statistics of PC-3-shSOX4 or the control cell-inoculated xenografts. **(D)** Representative IHC staining of SOX4, PCK2, NSE, SYP and Ki67 in tissues from the DU-145-shSOX4 or the control cell-inoculated xenografts. Scale Bar: 100 μm. **(E)** Representative IHC staining of SOX4, PCK2, NSE, SYP and Ki67 in tissues from the PC-3-shSOX4 or the control cell-inoculated xenografts. Scale Bar: 100 μm. Two-tailed Student’s t-test was used for statistical analysis: ns, not significant; *, *p* < 0.05; **, *p* < 0.01. Data are presented as means ± SD, *n* = 6

To evaluate the effect of SOX4 on tumor growth in vivo, we established a xenograft mouse model in castrated nude mice by inoculating DU-145 or PC-3 shSOX4 cells (Fig. 7A). As shown in Fig. 7B, tumor growth was observed in all of the mice (*n* = 6) inoculated with the control cells, while 4 of 6 mice were observed with limited tumor growth after inoculation of the DU-145-shSOX4 cells. As expected, the weight of the tumor in the DU-145-shSOX4 group was significantly lower compared to that of the control group. Similarly, SOX4 knockdown in PC-3 cells also inhibited the tumor growth in vivo (Fig. 7C). By IHC assay, we confirmed that SOX4 and PCK2 expression were repressed, along with the decreased expression of NE marker genes and Ki67 in the inoculated cells in vivo (Fig. 7D and E). Together, these results suggest that the knockdown of SOX4 effectively inhibits tumor growth as well as NE markers expression in vivo.

## Discussion

Although NE trans-differentiation has been widely reported as one of the most important causes of resistance to ARPI therapy in CRPC patients, the underlying mechanism regarding downstream signaling transduction and upstream regulation remains unclear [42]. In this study, we screened public databases and identified SOX4 as an upregulated gene in NEPC. We demonstrate gain of function and loss of function evidence that SOX4 promotes NE trans-differentiation.

While several downstream pathways including PI3K, Wnt and Sonic Hedgehog have been reported to be involved in the promotion of NE trans-differentiation [43–45], our study reveals that dysregulation of the metabolism reprogramming also plays an important role in promoting NE trans-differentiation. Specifically, our findings indicate that elevated expression of the pluripotent transcription factor SOX4 enhances aerobic glycolysis to promote NE trans-differentiation, which is evidenced by the increased intake of glucose and the increased production of lactate and pyruvic acid, as well as by upregulation of the expression of NE marker genes and downregulation of the AR expression as target genes of SOX4. Our hypothesis is supported by our previous studies. First, we previously showed Numb/Parkin pathway induces NE trans-differentiation via metabolic regulation of histone lactylation [46]. Second, we also reported that in neuroendocrine lung and prostate cancers, the activation of ADORA2A signaling triggers proline synthesis and results in neuroendocrine-like profiling changes [14]. Moreover, experiments by others demonstrated that hypoxia-induced upregulation of HMGCS1 can enhance lipid metabolism reprogramming to promote NE trans-differentiation in pancreatic cancer [47]. Thus, our findings reinforce the notion that dysregulation of the metabolism reprogramming is deeply involved in the promotion of NE trans-differentiation.

It is worth mentioning that in our study we demonstrated that elevated expression of the pluripotent transcription factor SOX4 enhances aerobic glycolysis as a manifestation of its regulation on carbohydrate metabolism reprogramming. Recent reports indicate that SOX4 promotes tumor proliferation by regulating glycolysis through activation of AKT in melanoma cells [24], yet the role of SOX4 in carbohydrate metabolism reprogramming to regulate cell plasticity is still poorly understood. In particular, the direct downstream effector of SOX4 in regulating the carbohydrate metabolism reprogramming remains unclear. Notably, we identify PCK2 as a novel target of SOX4 and a major downstream effector for the regulation of aerobic glycolysis in this study. Although PURα and p53 have been reported to promote PCK2 expression [48, 49], we provide experimental evidence to reveal a direct regulation of SOX4 on PCK2 expression at the transcriptional level by ChIP-qPCR assay and luciferase assay. The present study demonstrates that upregulation of PCK2 enhances aerobic glycolysis to promote NE trans-differentiation, in addition to its previously reported function in inhibition of the TCA cycle to promote tumor initiation [50]. Taken together, all these findings indicate that SOX4 can regulate aerobic glycolysis to promote NE trans-differentiation via the SOX4/PCK2 signaling.

In summary, our findings indicate that high expression of SOX4 is associated with a poor prognosis in CRPC patients. Moreover, SOX4 regulates carbohydrate metabolism reprogramming by directly enhancing PCK2 transcription to promote NE trans-differentiation. Our study suggests that the SOX4/PCK2 axis elements might be novel therapeutic targets to block NE trans-differentiation.

### Electronic supplementary material

Below is the link to the electronic supplementary material.

Supplementary Material 1

Supplementary Material 2

## Data Availability

Data is provided within the manuscript or supplementary information files.

## Abbreviations

ADT: Androgen deprivation therapy
AR: Androgen receptor
ARPI: AR pathway inhibitor
ChIP: Chromatin immunoprecipitation assay
CRPC: Castration Resistant prostate cancer
GEO: Gene Expression Omnibus
IHC: Immunohistochemistry
KEGG: Kyoto Encyclopedia of Genes and Genomes
NEPC: Neuroendocrine prostate cancer
PRAD: Prostate cancer
TCGA: The Cancer Genome Atlas
