# Supplementary material for: Targeting SOX4/PCK2 signaling suppresses neuroendocrine trans-differentiation of castration-resistant prostate cancer
Source: Biol Direct. 2024 Jul 16;19:56. doi: 10.1186/s13062-024-00500-2 (PMC11251300; doi:10.1186/s13062-024-00500-2)
Supplement: Supplementary file 1 — Supplementary Material 1 [file 13062_2024_500_MOESM1_ESM.pdf]

Supplementary Figure 1

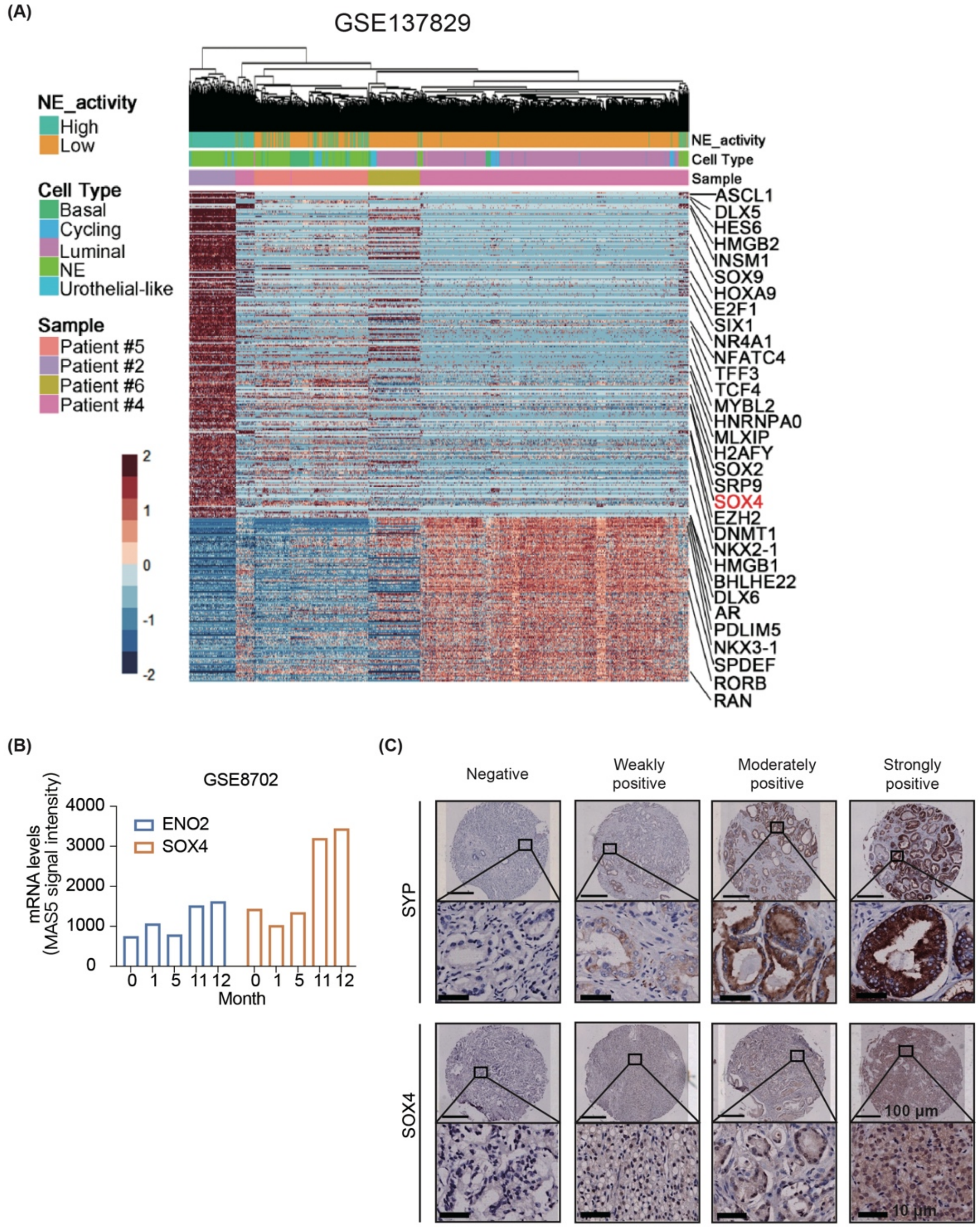

Fig. S1 The expression level of SOX4 in PCa cell lines and clinical patients.

(A) Bioinformatic analysis of a single cell RNA-seq dataset (GSE137829). **(B)** Trend of *SOX4* and neuroendocrine marker *ENO2* expression in androgen deprivation LNCaP cells from the GSE8702 dataset. **(C)** Representative IHC staining of SOX4 in a tissue microarray with

negative, weakly positive, moderately positive or strongly positive expression of neuroendocrine marker SYP. Upper panel scale bar: 100 μm, lower panel scale bar: 10 μm.

Supplementary Figure 2

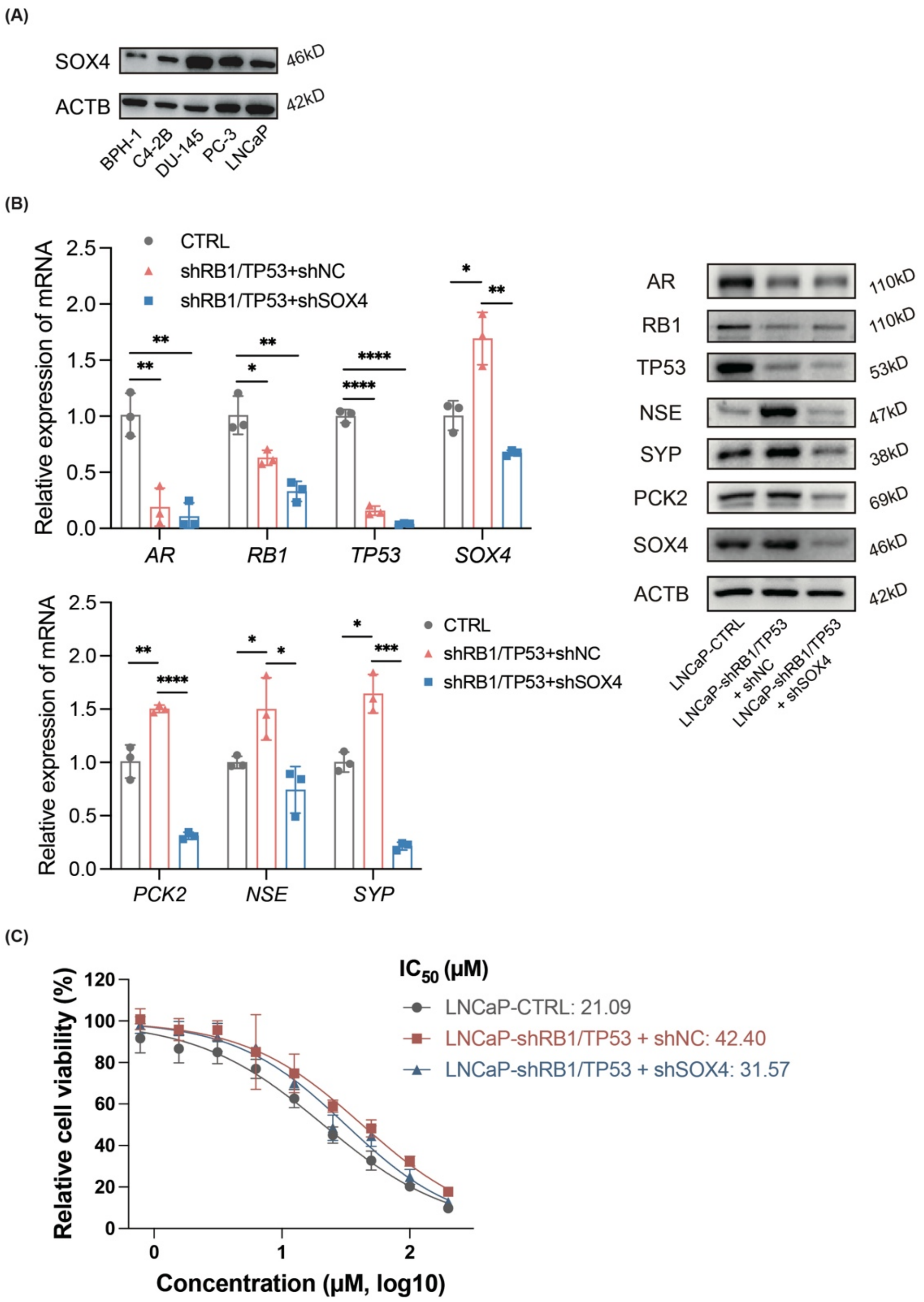

Fig. S2 Knockdown of SOX4 represses the NE trans-differentiation of PCa.

(A) Expression of SOX4 in different prostate cancer cell lines. **(B)** mRNA and protein expression of SOX4 in *SOX4*-knockdown LNCaP-shRB1/TP53 cells. **(C)** IC50 curves of enzalutamide resistance in LNCaP-shRB1/TP53 cells with or without SOX4 knockdown. Two-

tailed Student's t-test was used for statistical analysis: *, $p<0.05$; **, $p<0.01$; ***, $p<0.001$; ****, $p<0.0001$.

Supplementary Figure 3

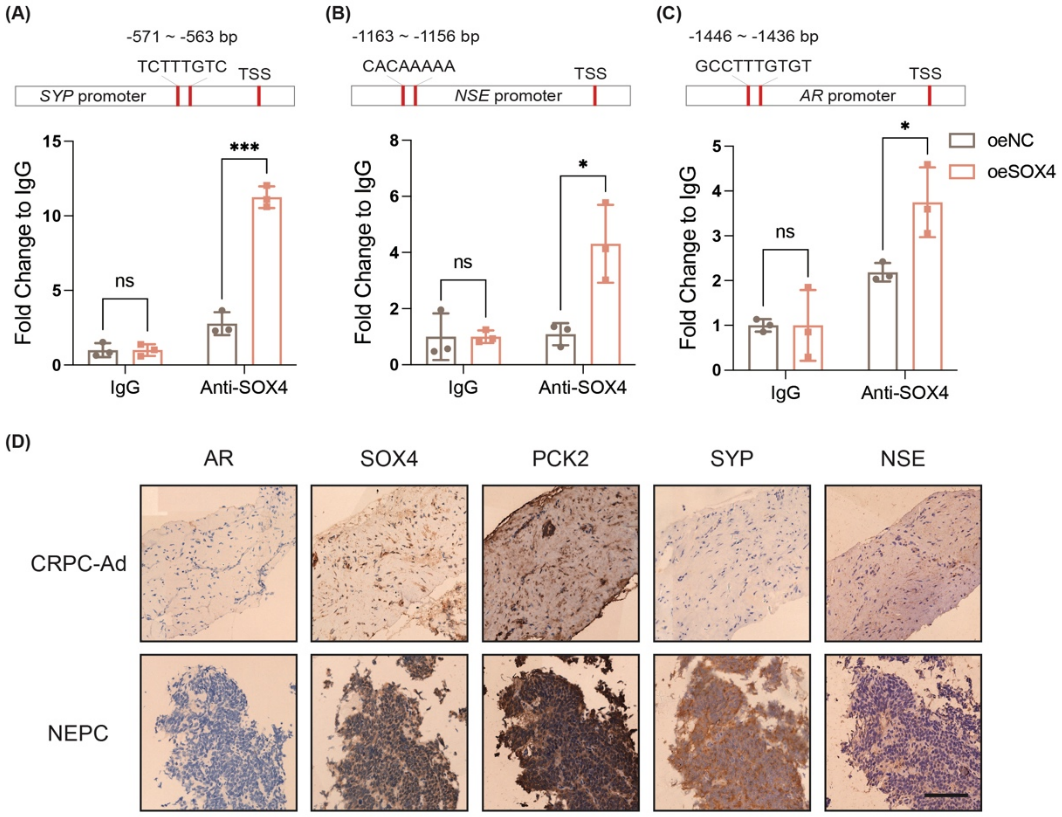

Fig. S3 SOX4 directly transcriptional regulates NE markers and AR expression.

(A) ChIP-qPCR assay of SOX4 binding on the promoter of SYP, NSE and AR in C4-2B cells.

(B) Representative IHC staining of SOX4 in clinical tumor sections of CRPC-Ad and NEPC patients. Scale bar: 100 µm. Two-tailed Student's t-test was used for statistical analysis: ns, not significant; *, p<0.05; **, p<0.01; ***, p<0.001.

Supplementary Figure 4

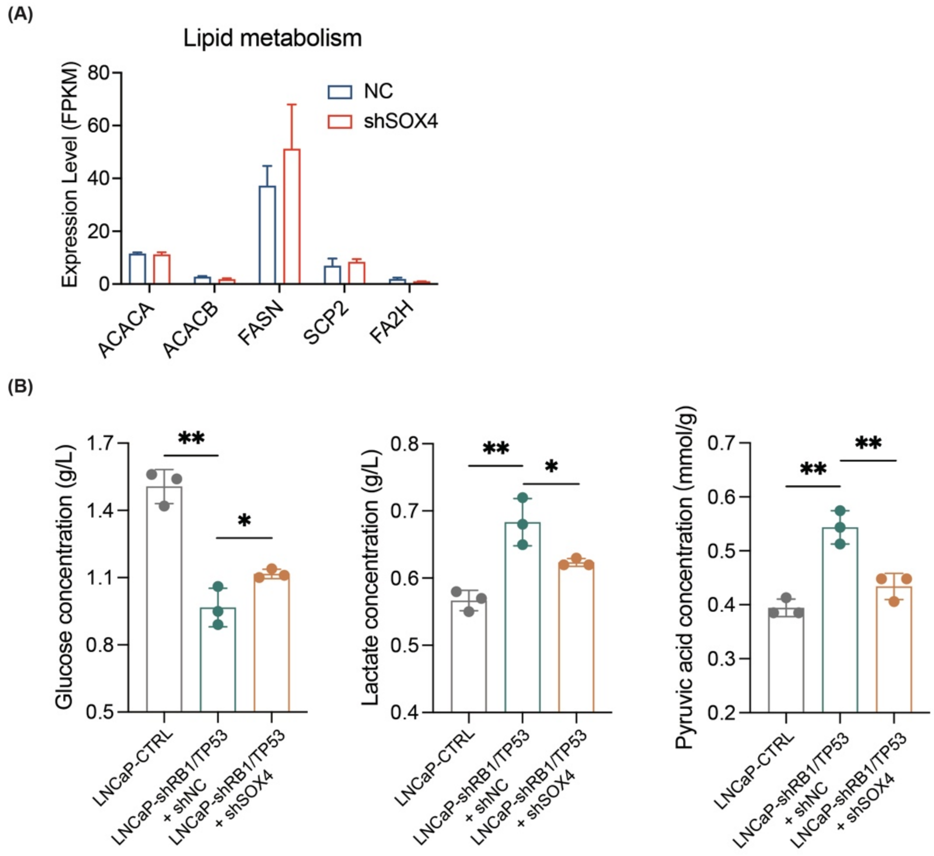

Fig. S4 SOX4 regulates carbohydrate metabolism reprogramming in PCa.

(A) Expression of key enzymes related to lipid metabolism in RNA-seq. **(B)** Knockdown SOX4 inhibited aerobic glycolysis in NEPC. Two-tailed Student's t-test was used for statistical analysis: ns, not significant; *, p<0.05; **, p<0.01.

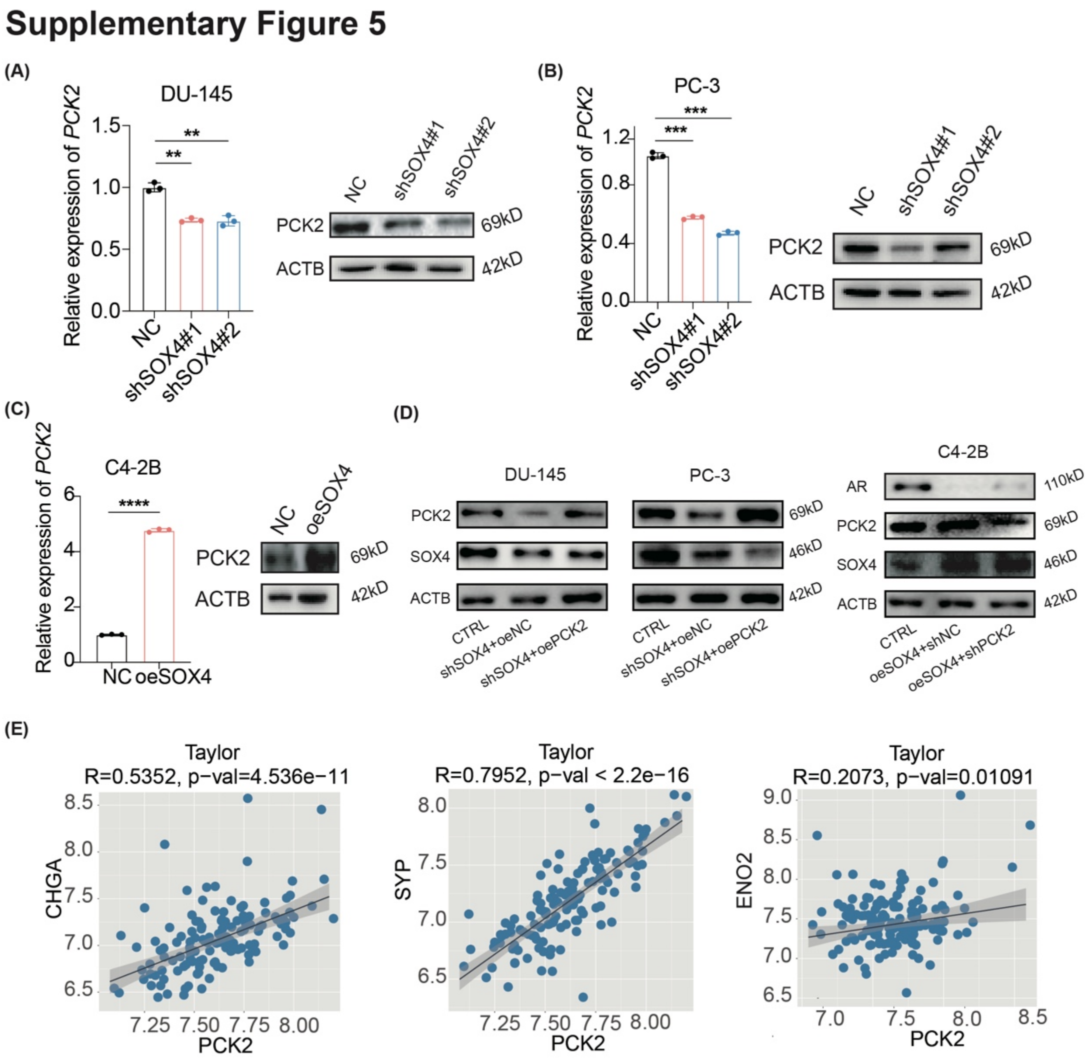

Fig. S5 SOX4 promotes NE trans-differentiation via regulating PCK2.

(A) mRNA and protein expression of PCK2 in *SOX4*-knockdown DU-145 cells. **(B)** mRNA and protein expression of PCK2 in *SOX4*-knockdown PC-3 cells. **(C)** mRNA and protein expression of PCK2 in *SOX4*-overexpressed C4-2B cells. **(D)** Verification of construction of PCK2 overexpression in DU-145-shSOX4 and PC-3-shSOX4 cell lines and PCK2 knockdown in C4-2B-oeSOX4 cell lines. **(E)** Correlation analysis between PCK2 and CHGA, SYP and NSE in the Taylor dataset. Two-tailed Student's t-test was used for statistical analysis: *, p<0.05; **, p<0.01; ***, p<0.001; ****, p<0.0001.
